# Modeling indoor TV/screen viewing and adult physical and mental health: Health Survey for England, 2012

**DOI:** 10.1007/s11356-016-6354-5

**Published:** 2016-03-05

**Authors:** Ivy Shiue

**Affiliations:** Faculty of Health and Life Sciences, Northumbria University, Newcastle upon Tyne, NE1 8ST England UK; Owens Institute for Behavioral Research, University of Georgia, Athens, GA USA; Alzheimer Scotland Dementia Research Centre, University of Edinburgh, Edinburgh, UK

**Keywords:** Chronic disease, Mental health, Screen, Television, Indoor radiation, Sedentary behavior, Cognitive performance, Self-rated health

## Abstract

The aim of the present study was to model indoor TV/screen viewing and a series of adult health conditions and cognitive performance in a country-wide, population-based setting in recent years. Data was retrieved from Health Survey for England, 2012. Information on demographics, lifestyle factors, self-reported health conditions, and TV and/or screen watching hours in adults was collected by household interviews. Chi-square test and survey-weighted logistic and multi-nominal modeling were performed. Of 8114 English adults aged 18–98, 4138 people (51.1 %) watched TV and/or screen daily for 2 h or more on average. Two thousand five-hundred people (30.9 %) watched for 3 h or more. TV and/or screening watching for 2+ hours was associated with endocrine or metabolic disorders, diabetes, mental disorders (including poor scores in General Health Questionnaire and Warwick-Edinburgh Mental Well-being Scale), nervous system disorders, eye complaints, circulatory system disorders, respiratory system disorders, musculoskeletal system disorders, and self-rated health. TV and/or screen watching for 3+ hours was associated with digestive disorders and clotting disorder. TV and/or screen watching for 5+ hours was associated with cancer. TV and/or screen watching for 6+, 8+, or 11+ hours was associated with bladder disease, genito-urinary system disorders or bowel disease, respectively. There were no risk associations (within 20 h) found with ear complaints, infectious disease, and blood system disorders. Future educational and public health programs minimizing TV and/or screen viewing in order to protect from physical inactivity and X-radiation might be needed while research on the combined effect of physical inactivity and X-radiation should be explored.

## Introduction

### Evidence before this study

Exposure to radiation has been found to increase blood-brain barrier (Nittby et al. 2009), and previous research mostly used regional electromagnetism transmission stations to measure the exposure, which are in the outdoor setting (Sirav and Seyhan 2009; Burch et al. 2006). This has left the knowledge gap in understanding the link of environmental exposure to indoor radiation and human health (Tofani et al. 1985). TV and/or screen viewing might be one of the sources, although likely being chronic rather than acute.

### Knowledge gap

The associations of the duration of TV and/or screen viewing, which could emit X-radiation however low frequency, and chronic health conditions of specific types and/or symptoms in adults and the very old have been less paid attention to, compared to that in children and adolescents. Methodologically, previous studies tended to correlate 2, 3, or 4 h with health conditions. In other words, effects of longer hours of TV and/or screen watching have not been examined yet.

### Study aim

Following this context, therefore, the aim of the present study was to model indoor TV/screen viewing and a series of adult health conditions including both physical and mental health using a unique statistical approach in an independent data set in a country-wide and population-based setting in recent years. It was also assumed that the study participants have had constant TV and/or screen viewing patterns over time.

## Methods

### Study sample

As described elsewhere in detail, Health Survey for England (more detail via http://www.hscic.gov.uk/article/3741/Health-Survey-for-England-Health-social-care-and-lifestyles) has been a country-wide, population-based, multi-year study since 1991. It is a major monitoring tool looking at the health of people who have resided in England. It is used by the British Government to plan health services and make important policy decisions that have an impact on its citizens. It includes information on individual physical health, lifestyle behaviors, social care, physical measures, mental health, and wellbeing. Participants are selected using a random probability sample. The survey design ensures that every address in England has an equal chance of being included in the survey each year and the results are representative of the population living in private households (more details via http://www.natcen.ac.uk/our-research/research/health-survey-for-england/). Data was collected by household interviews. In the current analysis, the most recent study wave in 2012 with the available data on demographics, lifestyle factors, self-reported health conditions, and TV and/or screen watching hours was selected.

### Variables and analyses

In the first step of data analysis, associations between TV and/or screen watching hours (x variable; Question: How much time did you spend sitting down watching TV, including DVDs and videos?) and a series of self-reported health conditions (y variables) in English adults aged 18–98 were assessed. Health conditions included endocrine or metabolic disorders, diabetes, mental disorders, nervous system disorders, eye complaints, ear complaints, circulatory system disorders, respiratory system disorders, musculoskeletal system disorders, infectious disease, blood system disorders and self-rated health, digestive disorders, clotting disorder, bladder disease, genito-urinary system disorder, or bowel disease (Question: Do you suffer from…?). In the second step, associations between TV and/or screen watching hours and English adult mental health using General Health Questionnaire 12-item (GHQ-12) and The Warwick-Edinburgh Mental Well-being Scale (WEMWBS) were examined. Of note, GHQ-12 has been utilized to assess subject’s mental health and psychological distress status (Goldberg 1978), and the WEMWBS is a scale of 14 positively worded items, with five response categories, for assessing a population’s mental wellbeing (more details via http://www.healthscotland.com/scotlands-health/population/Measuring-positive-mental-health.aspx). Both of them have been used and validated in many study populations over the last decades (Farmer and Harvey 1975; Clarke et al. 2011).

Covariates including age, sex, body mass index, hypertension, serum cotinine, and physical activity level were adjusted in the modeling. Effects were estimated by using odds ratios (OR) or relative risk ratios (RRR) together with 95 % confidence intervals (CI), depending on the outcome variables being binary or categorical. All the statistical models were weighted for the sampling and survey design. Since the study variable of TV and/or screen watching hours is ordinal, being 1 to 20 h, statistical analyses were executed repeatedly from 1 to 20 h in order to find out how many hours of TV and/or screen watching could and to what extent increase the risk of different adult chronic health conditions. Statistical software STATA version 13.0 (STATA, College Station, Texas, USA) was used to perform all the statistical analyses. The results were therefore listed in the corresponding tables accordingly. Of note, only statistically significant associations were shown.

### Ethics considerations

Since there were only secondary data analyses employed without any participant personal information identified by extracting statistical data from the study website in the present study, no further ethics approval for conducting the present study was required (more details via http://www.ethicsguidebook.ac.uk/Secondary-analysis-106).

## Results

### Descriptive statistics

Of all the 8114 English adults aged 18–98, 4138 people (51.1 %) watched TV and/or screen daily for 2 h or more on average. Two thousand five-hundred people (30.9 %) watched for 3 h or more. Seven hundred seventy-one people (9.5 %) watched for 5 h or more. Four hundred five people (5.0 %) watched for 6 h or more. One hundred sixty-two people (2.0 %) watched for 8 h or more. Sixty-one people (0.8 %) watched for 11 h or more.

### Analytical statistics

Table 1 shows associations between TV and/or screen watching hours and self-reported health conditions in English adults. Reporting TV and/or screen watching for 2+ hours was associated with endocrine or metabolic disorders, diabetes, mental disorders, nervous system disorders, eye complaints, circulatory system disorders, respiratory system disorders, musculoskeletal system disorders, and self-rated health. Reporting TV and/or screen watching for 3+ hours was associated with digestive disorders and clotting disorder. Reporting TV and/or screen watching for 5+ hours was associated with cancer while reporting TV and/or screen watching for 6+, 8+ or 11+ hours was associated with bladder disease, genito-urinary system disorders, or bowel disease, respectively.

**Table 1:** Associations between TV/screen watching duration and common adult health conditions (*n* = 8114; aged 18–98)

	<2 h (*n* = 3961, 48.9 %)	>=2 h (*n* = 4138, 51.1 %)	OR (95 % CI)^a^	*P* value
Endocrine/metabolic				
No	3706 (50.8 %)	3587 (49.2 %)	1.00	
Yes	251 (31.5 %)	547 (68.6 %)	1.45 (1.20–1.76)	<0.001
Diabetes				
No	3777 (50.6 %)	3695 (49.5 %)	1.00	
Yes	183 (29.5 %)	438 (70.5 %)	1.60 (1.30–1.98)	<0.001
Nervous system				
No	3849 (49.5 %)	3921 (50.5 %)	1.00	
Yes	108 (33.6 %)	213 (66.4 %)	1.68 (1.26–2.24)	<0.001
Eye complaints				
No	3892 (49.6 %)	3960 (50.4 %)	1.00	
Yes	65 (27.2 %)	174 (72.8 %)	1.48 (1.06–2.07)	0.021
Heart/Circulatory system				
No	3651 (51.7 %)	3417 (48.3 %)	1.00	
Yes	306 (29.9 %)	7171 (70.1 %)	1.39 (1.17–1.65)	<0.001
Respiratory system				
No	3711 (49.7 %)	3764 (50.4 %)	1.00	
Yes	246 (39.9 %)	370 (60.1 %)	1.21 (1.00–1.46)	0.052
Musculoskeletal system				
No	3475 (52.0 %)	3205 (48.0 %)	1.00	
Yes	482 (34.2 %)	9292 (65.8 %)	1.35 (1.17–1.55)	<0.001
	<3 h (*n* = 5599, 69.1 %)	>=3 h (*n* = 2500, 30.9 %)	OR (95 % CI)^a^	*P* value
Mental disorders				
No	5319 (69.6 %)	2319 (30.4 %)	1.00	
Yes	274 (60.5 %)	179 (39.5 %)	1.63 (1.30–2.04)	<0.001
Digestive system				
No	5374 (69.9 %)	2320 (30.2 %)	1.00	
Yes	219 (55.2 %)	178 (44.8 %)	1.42 (1.14–1.78)	0.002
Clotting disorder				
No	3538 (66.5 %)	1628 (31.5 %)	1.00	
Yes	63 (43.2 %)	83 (56.9 %)	1.56 (1.08–2.25)	0.017
	<5 h (*n* = 7328, 90.5 %)	> = 5 h (*n* = 771, 9.5 %)	OR (95 % CI)^a^	*P* value
Cancer				
No	7189 (90.8 %)	733 (9.3 %)	1.00	
Yes	132 (78.1 %)	37 (21.9 %)	1.63 (1.07–2.47)	0.022
	<6 h (*n* = 7694, 95.0 %)	>=6 h (*n* = 405, 5.0 %)	OR (95 % CI)^a^	*P* value
Bladder disease				
No	1244 (91.9 %)	110 (8.1 %)	1.00	
Yes	361 (88.1 %)	49 (12.0 %)	1.52 (1.05–2.19)	0.026
	<8 h (*n* = 7937, 98.0 %)	>=8 h (*n* = 162, 2.0 %)	OR (95 % CI)^a^	*P* value
Genito-urinary system				
No	7766 (98.1 %)	153 (1.9 %)	1.00	
Yes	164 (95.4 %)	8 (4.7 %)	2.55 (1.13–5.79)	0.025
	<11 h (*n* = 8038, 99.2 %)	>=11 h (*n* = 61, 0.8 %)	OR (95 % CI)^a^	*P* value
Bowel disease				
No	1622 (99.1 %)	15 (0.9 %)	1.00	
Yes	128 (97.0 %)	4 (3.0 %)	3.71 (1.02–13.45)	0.046

^a^Adjusted for age, sex, body mass index, hypertension, smoking status, indoor second-hand smoking, and physical activity

Tables 2 and 3 present associations between TV and/or screen watching pattern and English adult mental health by GHQ-12 and WEMWBS, respectively. Reporting watching TV and/or screen for 2 h or more was associated with poor GHQ-12 scores. Specific symptoms included lost sleep over worry, felt not playing useful part in things, felt incapable of making decisions, felt could not overcome difficulties, unable to enjoy day-to-day activities, been unable to face problems, been feeling unhappy and depressed, been losing confidence in self, been thinking of self as worthless, not feeling optimistic about the future, not feeling useful, not feeling interested in other people, not dealing with problems well, not feeling good about myself, not feeling confident, not able to make up my own mind about things, not interested in new things, and not feeling cheerful. Other symptoms included unable to concentrate, felt constantly under strain, no energy to spare, not feeling relaxed, not thinking clearly, not feeling close to other people, and not feeling loved when people reported watching TV and/or screen for 3 h or more.

**Table 2:** Associations between daily TV/screen watching and adult mental health by General Health Questionnaire

	<2 h (*n* = 3961, 48.9 %)	>=2 h (*n* = 4138, 51.1 %)	RRR (95 % CI)^a^	*P* value
Self-rated health				
Good to very good	3247 (54.6 %)	2696 (45.4 %)	1.00	
Fair	546 (35.6 %)	990 (64.5 %)	1.67 (1.47–1.91)	<0.001
Poor to very poor	168 (27.2 %)	450 (72.8 %)	2.43 (1.96–3.01)	<0.001
GHQ-12				
Score 0	2169 (49.5 %)	2209 (50.5 %)	1.00	
Score 1–3	841 (50.9 %)	812 (49.1 %)	1.00 (0.88–1.14)	0.971
Score 4+	482 (45.0 %)	589 (55.0 %)	1.23 (1.07–1.43)	0.005
Lost sleep over worry				
Normal	2945 (49.4 %)	3012 (50.6 %)	1.00	
More than usual	604 (47.3 %)	673 (52.7 %)	1.23 (1.07–1.42)	0.004
Felt playing useful part in things				
Normal	3212 (50.1 %)	3197 (49.9 %)	1.00	
Less than usual	316 (40.6 %)	463 (59.4 %)	1.23 (1.07–1.51)	0.007
Felt capable of making decisions				
Normal	3332 (49.7 %)	3369 (50.3 %)	1.00	
Less than usual	219 (40.9 %)	317 (59.1 %)	1.28 (1.05–1.57)	0.015
Felt could not overcome difficulties				
Normal	3213 (49.7 %)	3251 (50.3 %)	1.00	
More than usual	336 (44.0 %)	428 (56.0 %)	1.29 (1.09–1.53)	0.003
Been able to face problems				
Normal	3294 (49.9 %)	3303 (50.1 %)	1.00	
Less than usual	264 (40.2 %)	393 (59.8 %)	1.38 (1.15–1.65)	<0.001
Been losing confidence in self				
Normal	3145 (50.0 %)	3151 (50.1 %)	1.00	
More than usual	411 (43.0 %)	546 (57.1 %)	1.39 (1.20–1.62)	<0.001
Been thinking of self as worthless				
Normal	3338 (49.8 %)	3372 (50.3 %)	1.00	
More than usual	216 (39.9 %)	326 (60.2 %)	1.55 (1.26–1.90)	<0.001
Been feeling reasonably happy				
Normal	3201 (49.4 %)	3275 (50.6 %)	1.00	
Less than usual	355 (45.8 %)	420 (54.2 %)	1.19 (1.00–1.40)	0.048
	<3 h (*n* = 5599, 69.1 %)	>=3 h (*n* = 2500, 30.9 %)	OR (95 % CI)^a^	*P* value
Able to concentrate				
Normal	4496 (70.6 %)	1876 (29.4 %)	1.00	
Less than usual	533 (61.8 %)	329 (38.2 %)	1.43 (1.21–1.68)	<0.001
Felt constantly under strain				
Normal	4094 (69.8 %)	1771 (30.2 %)	1.00	
More than usual	935 (68.6 %)	428 (31.4 %)	1.18 (1.02–1.37)	0.023
Able to enjoy day-to-day activities				
Normal	4355 (71.7 %)	1770 (28.9 %)	1.00	
Less than usual	688 (60.4 %)	451 (39.6 %)	1.47 (1.27–1.70)	<0.001
Been feeling unhappy and depressed				
Normal	4326 (70.4 %)	1818 (29.6 %)	1.00	
More than usual	713 (64.4 %)	394 (35.6 %)	1.37 (1.19–1.59)	<0.001

^a^Adjusted for age, sex, body mass index, hypertension, smoking status, indoor second-hand smoking, and physical activity

**Table 3:** Associations between daily TV/screen watching and adult mental health by Warwick-Edinburgh Mental Well-being Scale

	<2 h (*n* = 3961, 48.9 %)	>=2 h (*n* = 4138, 51.1 %)	OR (95 % CI)^a^	*P* value
Been feeling optimistic about the future				
Normal	2203 (48.7 %)	2325 (51.4 %)	1.00	
Less than sometimes	221 (37.7 %)	366 (62.4 %)	1.37 (1.12–1.68)	0.002
Been feeling useful				
Normal	2282 (48.3 %)	2444 (51.7 %)	1.00	
Less than sometimes	141 (36.1 %)	250 (63.9 %)	1.50 (1.18–1.89)	0.001
Been feeling interested in other people				
Normal	2265 (48.4 %)	2420 (51.7 %)	1.00	
Less than sometimes	151 (37.4 %)	253 (62.6 %)	1.56 (1.24–1.96)	<0.001
Been dealing with problems well				
Normal	2294 (47.9 %)	2492 (52.1 %)	1.00	
Less than sometimes	132 (38.9 %)	207 (61.1 %)	1.36 (1.06–1.74)	0.014
Been feeling good about myself				
Normal	2230 (48.1 %)	2408 (51.9 %)	1.00	
Less than sometimes	194 (39.6 %)	296 (60.4 %)	1.43 (1.14–1.79)	0.002
Been feeling confident				
Normal	2236 (48.2 %)	2403 (51.8 %)	1.00	
Less than sometimes	187 (39.1 %)	291 (60.9 %)	1.49 (1.20–1.86)	<0.001
Been able to make up my own mind about things				
Normal	2348 (47.6 %)	2587 (52.4 %)	1.00	
More than sometimes	79 (39.5 %)	121 (60.5 %)	1.42 (1.03–1.97)	0.033
Been interested in new things				
Normal	2266 (48.5 %)	2411 (51.6 %)	1.00	
More than sometimes	167 (35.8 %)	300 (64.2 %)	1.53 (1.22–1.92)	<0.001
Been feeling cheerful				
Normal	2297 (47.5 %)	2537 (52.5 %)	1.00	
More than sometimes	134 (43.2 %)	176 (56.8 %)	1.42 (1.10–1.83)	0.007
	<3 h (*n* = 5599, 69.1 %)	>=3 h (*n* = 2500, 30.9 %)	OR (95 % CI)^a^	*P* value
I have had energy to spare				
Normal	2746 (71.2 %)	1109 (28.8 %)	1.00	
Less than sometimes	748 (59.2 %)	516 (40.8 %)	1.49 (1.29–1.71)	<0.001
Been feeling relaxed				
Normal	3104 (69.0 %)	1395 (31.0 %)	1.00	
Less than sometimes	375 (63.1 %)	219 (36.9 %)	1.54 (1.26–1.90)	<0.001
Been thinking clearly				
Normal	3372 (68.7 %)	1535 (31.3 %)	1.00	
Less than sometimes	128 (56.9 %)	97 (43.1 %)	1.75 (1.28–2.39)	<0.001
Been feeling close to other people				
Normal	3269 (68.8 %)	1482 (31.2 %)	1.00	
Less than sometimes	219 (60.3 %)	144 (39.7 %)	1.63 (1.26–2.12)	<0.001
	<4 h (*n* = 6721, 83.0 %)	>=4 h (*n* = 1378, 17.1 %)	OR (95 % CI)^a^	*P* value
Been feeling loved				
Normal	4109 (83.2 %)	809 (16.8 %)	1.00	
Less than sometimes	205 (70.7 %)	85 (29.3 %)	1.97 (1.46–2.66)	<0.001

^a^Adjusted for age, sex, body mass index, hypertension, smoking status, indoor second-hand smoking, and physical activity

## Discussion

### TV and/or screen watching and physical health

Increased TV watching may be a sign of environmental dependency and therefore leads to loss of personal autonomy such that a person’s environment almost entirely controls their actions (Shin et al. 2013). In American black women aged 21–69, TV watching was associated with an increased type 2 diabetes risk: The incidence rate ratio was 1.86 (95 % CI 1.54–2.24) for >=5 h relative to <1 h of television per day, independent of physical activity (Krishnan et al. 2009). Similar results were obtained in American men aged 40–75 (Hu et al. 2001), the relative risks of diabetes across categories of average hours spent watching TV per week (0–1, 2–10, 11–20, 21–40, and >40) were 1.00, 1.66, 1.64, 2.16, and 2.87, respectively (*P* for trend <0.001). In English older adults, TV viewing time was associated with incident diabetes mellitus at 2-year follow-up (≥6 h/day compared with <2 h/day; OR 4.27, 95 % CI 1.69–10.77), although the association was attenuated to the null in final adjusted models that included body mass index (Smith and Hamer 2014). Compared with those in the lowest quartile, the ORs of the metabolic syndrome in the highest quartile of TV viewing time were 1.42 (95 % CI 0.93–2.15) for Australian men and 1.42 (95 % CI 1.01–2.01) for women aged 60 and above (Gardiner et al. 2011). In 10 European countries, adults aged 45–79 were observed to have increased risk of total cardiovascular disease or coronary heart disease in those who had 1 h or more TV watching per day (Wijndaele et al. 2011). Moreover, TV viewing time was found to be inversely associated with both total body lean mass (*P* = 0.05) and leg lean mass (*P* < 0.01) in Australian adults aged 60–86 (Gianoudis et al. 2014). In a very recent metal-analysis, it was concluded that TV watching time could have increased risks of colon cancer, endometrial cancer, lung cancer but not cancers of the breast, rectum, ovaries, prostate, stomach, esophagus, testes, renal cell, and non-Hodgkin lymphoma (Schmid and Leitzmann 2014). These studies tended to have various measurements and statistical modeling with various sizes of study populations that might need validity checking. Literature on other included physical health conditions was very scarce. Therefore, it was not possible to compare and discuss the implications.

### TV and/or screen watching and mental health

Although the relationship of TV viewing and mental health such as depression might not be uncommon (Teychenne et al. 2010), research focusing on adults is still limited. Previously (Hamer et al. 2010), it was observed that Scottish adults with the highest TV and/or screening watching time level (>4 h/day) had an increase in GHQ-12 score of 0.28 (95 % CI 0.05–0.51), compared with those with the lowest level (< or =2 h/day). In Australian adults aged 25 and above, each 1-h/day increment in TV viewing time was associated with lower physical (−0.56 [95 % CI −0.77, −0.34]) and mental (−0.41 [−0.70, −0.12]) component summary scores and vitality (−0.51 [−0.81, −0.21]) using SF-36 questionnaire (Hetsroni 2012). There is also a lack of research looking into how TV and/or screening watching might affect daily cognitive function in adults. For example, previous psychological research observed that love styles could have also been influenced by TV watching and further affect one’s emotional and/or behavioral problems (Hetsroni and Lowenstein 2013). Such situation might give an alarm on future potential criminal and/or victimization behaviors (Dempsey et al. 2014). In the present study, the large quantitative research evidence showed that more TV and/or screening (more than 3 h) was related to less feeling of being loved in addition to unhappiness and other negative thoughts. This could come with the less clear thinking and close relationship with others as co-effects, as shown in the results.

### X-radiation exposure in the household

If we believe that in every English household the X-radiation is limited to the standard as required starting from the end of 2012 (more details via http://en.wikipedia.org/wiki/Analogue_terrestrial_television_in_the_United_Kingdom#405_line_system), the exposure per day shall be between 45 Megahertz (MHz) and 847.25 MHz. If we assume that on average in every English household the wavelength from using TV is 446.125 MHz per hour, by using the formula (45 + 847.25)/2, this could mean that roughly the English adults were exposed to 446.125 to 8922.5 MHz (1–20 h) per day, although distance to TV/screen might need to be included in the equation as well. Likely, they could have started to develop health problems when it reached 892.25 MHz and above (2+ hours). However, this estimation has excluded the time on the use of other technology with screens that could also emit varying degrees of X-radiation.

### Strengths and limitations

The present study has a few strengths. Firstly, it lied in its very large and representative study sample, being country-wide and population-based, and in recent years. Secondly, it was also the first time to model how TV and/or screen watching hours were correlated with different adult chronic health conditions in England. Thirdly, the study variable of TV and/or screen watching was not binary, being yes or no, but ordinal by presenting in hours. Therefore, it was available to examine how many hours of TV and/or screen watching (up to 20 h) could be linked with different chronic health conditions.

However, there are also a few limitations that cannot be ignored. First, it was unable to include all the subtypes of each health condition and the objective measurement of TV and/or screening viewing due to the limitation of the current dataset. Second, it was also unable to examine if and how engaging with indoor exercise during TV and/or screen watching intervals might help lessen the risk effect on human health and well-being (Mekary et al. 2013). Third, the causality cannot be established due to the cross-sectional study design in nature. There might be reverse causation bias that was unavoidable. Specifically, whether TV and/or screen viewing habits and patterns could have changed after certain health events (this could be one-time point or continuous) was unknown. For example, it was recently observed that people with mental disorders (e.g., dysthymia, panic disorder, and agoraphobia) could have spent more time viewing TV than people without (de Wit et al. 2011). Fourth, health conditions were only self-reported in the survey but not cross-checked with hospital admissions by physician diagnosis. Therefore, there might be some underestimates or overestimates in the disease prevalence. Still, there could be diagnosis or classification bias in hospital admissions resulting in diagnosis avoidance or over-diagnosis (Burgard and Chen 2014; Eggers et al. 2009; Minasian et al. 2013). Taken together, future studies keeping the strengths and overcoming the limitations mentioned above in a longitudinal or experimental approach to confirm or refute the current findings would be recommended.

### Research, practice, and policy implications

In sum, over half of English adults viewed TV and/or screen for 2+ hours daily while almost one-third English adults viewed TV and/or screen daily for 3+ hours daily. TV and/or screening viewing for 2+ hours was observed to be associated with endocrine or metabolic disorders, diabetes, mental disorders, nervous system disorders, eye complaints, circulatory system disorders, respiratory system disorders, musculoskeletal system disorders, and self-rated health. TV and/or screen viewing for 3+ hours was found to be associated with digestive disorders and clotting disorder. Moreover, TV and/or screen viewing for 5+ hours was associated with cancer and TV and/or screen viewing for 6+, 8+, or 11+ hours was associated with bladder disease, genito-urinary system disorders, or bowel disease, respectively.

Too much TV and/or screen viewing is harmful to both physical and mental health. Recent research has also observed the potential risk effect on DNA damage in children (Himmetoglu et al. 2015). For future research, a longitudinal or experimental approach to confirm or refute the current findings while examining the role of TV/screening viewing in any illness recovery and/or family relationship to form the healthy and balanced lifestyle would be suggested. In addition, examination on the accumulation of indoor X-radiation in the household over time in addition to the outdoor radiation around the household to fully understand the long-term health effects for occupants should be considered. For clinical practice and policy, future educational, public health, and nursing programs minimizing TV and/or screen viewing alongside regulation on X-radiation at <=0.5 milliroentgen per hour (Luketina 1975) in order to protect, sustain, and then optimize human health might need to be encouraged (Hietanen and Hoikkala 1990; Setter et al. 1969).
